# Inhibitory Circuits of the Sensorimotor Network are Not Modulated By Cerebellar Transcranial Direct Current Stimulation in Isolated Cervical Dystonia

**DOI:** 10.1007/s12311-026-02024-z

**Published:** 2026-05-16

**Authors:** Franca Peemöller, Kai Grimm, Ronak Rashedi, Mathias Gelderblom, Simone Zittel

**Affiliations:** Department of Neurology, University Medical Center Hamburg-Eppendorf, Martinistr. 52, Hamburg, 20246 Germany

**Keywords:** Cervical dystonia, Cerebellum, Transcranial direct current stimulation, Transcranial magnetic stimulation, Intracortical inhibition, Afferent inhibition, Cerebellar brain inhibition

## Abstract

Impaired cerebellar influence on motor cortical excitability and plasticity has been reported in cervical dystonia (CD) patients, accompanied by the absence of cerebellar brain inhibition (CBI). Polarity-specific modulation of CBI in healthy individuals using cerebellar transcranial direct current stimulation (ctDCS) suggested that ctDCS could normalize abnormal cerebellar output and improve clinical symptom severity in CD. The objective of this study was to determine whether anodal or cathodal ctDCS can modulate neurophysiological parameters of cortico-cortical, cerebello-cortical or afferent inhibition and improve motor symptom severity in CD patients. Fifteen patients with isolated CD participated in a randomized, double-blinded crossover study consisting of three sessions of anodal, cathodal, or sham ctDCS. Before and after each intervention, motor symptom severity and inhibitory circuits of the sensorimotor network were investigated using transcranial magnetic stimulation (TMS), including short-interval intracortical inhibition (SICI), short-latency afferent inhibition (SAI) and CBI. Baseline TMS measurements showed inhibitory influence of SICI (p < 0.001) and SAI (p < 0.001) in CD patients, whereas CBI had no inhibitory effect (p = 0.281). The different ctDCS interventions caused no significant modulation in any of the inhibitory TMS paradigms investigated. Similarly, motor symptom severity remained unchanged after the ctDCS interventions. A single session of ctDCS was not effective to modulate inhibitory circuits of the sensorimotor network in isolated CD patients. Future studies focusing on repetitive ctDCS interventions or multifocal stimulation protocols are needed to assess the influence on network excitability and connectivity as well as on motor symptoms.

## Introduction

Dystonia is a movement disorder characterized by sustained or intermittent muscle contractions, leading to abnormal, often repetitive movements or postures [1]. Based on the distribution of affected body regions, dystonias can be classified as focal, segmental, multifocal or generalized. Among focal dystonias, cervical dystonia (CD) is the most common form, accounting for more than 40% of all dystonia cases [2]. CD is characterized by involuntary contractions of the neck and shoulder muscles, resulting in abnormal postures and movements of the head. First-line treatments for CD – most notably, regular botulinum toxin injections and physical therapy – may not always provide sufficient relief of symptoms or fail to offer lasting benefits [3, 4]. As a result, there is increasing interest in exploring new therapeutic approaches, including non-invasive brain stimulation (NIBS) techniques, to provide more sustained or targeted relief of symptoms.

The pathophysiological mechanisms underlying CD remain incompletely understood, which complicates the development of novel therapeutic approaches. Although CD is clinically characterized as a focal dystonia affecting the neck muscles, accumulating evidence from structural and functional neuroimaging, animal models, and neurophysiological studies established a complex network disorder model, involving widespread motor and sensorimotor circuits, with dysfunction in the basal ganglia, the brainstem, the sensorimotor cortex, and the cerebellum [5, 6]. In this context, alterations in motor cortical excitability were not restricted to clinically affected muscles in CD, but extended to distant body regions, including the upper limbs [7]. Clinical studies have demonstrated functional impairments in non-dystonic body parts in CD patients, such as altered fine motor control of the hands [8], and transcranial magnetic stimulation (TMS) studies using paired associative stimulation (PAS) have shown a loss of topographic specificity in CD, with facilitation in different hand muscles spreading beyond the targeted muscle [9, 10]. Together, these findings reflect a generalized disturbance of motor network organization in CD, providing the rationale for the assessment of motor evoked potentials (MEPs) in hand muscles, an approach that has been consistently applied in previous TMS studies in CD [11–13].

In the last years, the cerebellum has emerged as a critical component in the proposed dystonia network, e.g. human magnetic resonance imaging studies demonstrated altered cerebellar connectivity in CD patients [14], and neurophysiological studies suggested impaired cerebellar modulation of cortical excitability and plasticity [5, 12, 13]. Transcranial direct current stimulation (tDCS) is a NIBS technique that modulates neuronal excitability in a bidirectional manner by applying a weak electrical current via scalp electrodes. Depending on the polarity of stimulation, anodal tDCS increases and cathodal tDCS decreases neuronal excitability, with effects persisting beyond the stimulation period [15, 16]. Although traditionally applied over the motor cortex, tDCS targeting the cerebellum (ctDCS) has attracted increasing interest as a tool to assess functional aspects and motor network connectivity [17]. Given the pathophysiological role of the cerebellum in CD, ctDCS may be a suitable technique to modulate motor network alterations associated with this disease.

With regard to neurophysiological measures investigated in dystonia by TMS, increased excitability of the sensorimotor network has been frequently reported. Impaired intracortical inhibition of the primary motor cortex (M1), reduced short-latency afferent inhibition (SAI), and increased sensorimotor plasticity have emerged as key neurophysiological features, presumably contributing to excessive motor output and abnormal muscle contractions [5, 18, 19]. A particular neurophysiological marker to assess cerebello-thalamo-cortical (CTC) connectivity is cerebellar brain inhibition (CBI), in which the application of a conditioning stimulus (CS) over the cerebellum followed by a test stimulus (TS) over the contralateral M1 at short interstimulus intervals (ISIs) leads to a suppression of the MEP amplitude to approximately 50–80% compared to the TS alone [20–23]. Brighina et al. first demonstrated that CBI is markedly reduced or even absent in patients with CD and focal hand dystonia (FHD) [13], indicating an impaired inhibitory influence of the cerebellum on M1. As ctDCS induced a polarity-specific modulation of CBI in healthy individuals, with anodal stimulation increasing and cathodal stimulation decreasing CBI [24], ctDCS may have the potential to modulate altered CTC connectivity in CD.

Taken together, the previous findings formed the rationale for our study to assess the potential of anodal and cathodal ctDCS to effectively modulate specific neurophysiological markers of cortico-cortical, cerebello-cortical, and afferent inhibition, and to reduce clinical symptom severity in CD patients.

## Materials and Methods

### Study Participants

Patients were recruited from the outpatient clinic of the Department of Neurology at the University Medical Center Hamburg-Eppendorf. Inclusion criteria were (i) a clinical diagnosis of isolated CD, (ii) age ≥ 18 years, and (iii) the ability to consent to the study. All patients were receiving regular botulinum toxin injections and were investigated 9 to 12 weeks after their last injection to minimize potential influences on the measurements. Exclusion criteria included (i) any known neurological disorders other than CD, (ii) secondary dystonia, (iii) severe head tremor, (iv) medication affecting the central nervous system, and (v) contraindications to TMS such as metallic implants, pregnancy, or history of seizures [25]. Handedness was determined using the Edinburgh Handedness Inventory [26]. Before and after each session, motor symptom severity was examined and recorded on video for subsequent evaluation using the Toronto Western Spasmodic Torticollis Rating Scale (TWSTRS) [27]. All patients gave written informed consent in accordance with the Declaration of Helsinki and completed TMS safety questionnaires. The study was approved by the local ethics committee (PV5819-4119-BO-ff).

### Experimental Design

**Fig. 1:**
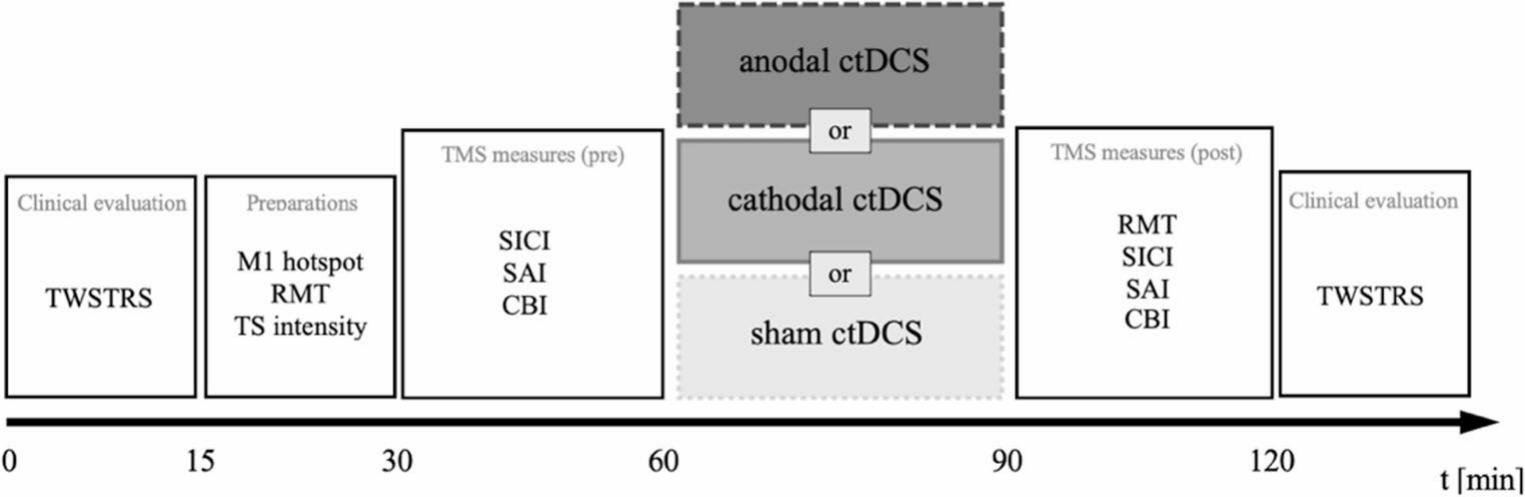
Experimental design of the study. The arrow indicates the timeline of the experiment in minutes. The mode of ctDCS (anodal, cathodal, sham) was randomized across three sessions. *TWSTRS* Toronto Western Spasmodic Torticollis Rating Scale, *M1* primary motor cortex, *RMT* resting motor threshold, *TS* test stimulus, *SICI* short-interval intracortical inhibition, *SAI* short-latency afferent inhibition, *CBI* cerebellar brain inhibition, *ctDCS* cerebellar transcranial direct current stimulation

The study consisted of three experimental sessions for each patient, which took place at intervals of one week. All sessions were conducted in the morning because circadian rhythm effects are known to influence cortical excitability [28]. In each session, patients underwent a different type of ctDCS intervention: anodal, cathodal, or sham stimulation (see Fig. 1). Both the subjects and the experimenter were blinded to the stimulation mode, and the trial order was randomized. Before and after each ctDCS intervention, TMS was used to assess the neurophysiological properties of the sensorimotor network using different paradigms. As a measure of global motor cortical excitability, the resting motor threshold (RMT) was determined. In addition, three specific TMS paradigms with known inhibitory effects on M1 were applied: short-interval intracortical inhibition (SICI), SAI, and CBI. Each session began and ended with a clinical evaluation of motor symptom severity using the the TWSTRS Part 1. This study was prospectively registered at *ClinicalTrials.gov* (Identifier: NCT07014384).

### TMS Setup and EMG Recordings

During the experiment, patients were seated comfortably in a chair with both arms resting on a pillow. They were instructed to relax their arms and to keep their eyes open throughout the whole experiment. TMS was applied using a BiStim^2^ stimulator combining two 200^2^ units (Magstim Co Ltd., Whitland, UK) for SICI and one or two 70 mm diameter figure-of-eight coils (Magstim Co Ltd., Whitland, UK) depending on the different TMS paradigms. For the stimulation of M1, the coil was positioned tangentially to the scalp with the handle pointing backwards and laterally at a 45-degree angle [29]. For the stimulation of the cerebellum during CBI, another 70 mm diameter figure-of-eight coil was positioned over the right cerebellar hemisphere 3 cm lateral and 1 cm caudal to the inion tangentially on the scalp, with the handle pointing upwards to induce upward current in the cerebellar cortex as described by Koch et al. [11].

Electromyography (EMG) recordings were obtained from the right first dorsal interosseus (FDI) muscle using disposable surface electrodes (20 × 25 mm, Ag-AgCl surface electrodes, Spes Medica S.p.A., Genoa, Italy) in a belly-tendon montage. EMG signals were amplified and filtered (20 Hz and 2 kHz) using a CED 1902 amplifier (Cambridge Electronic Design, Cambridge, UK). Signals were sampled at 5 kHz, digitized using a laboratory interface (Micro 1401; Cambridge Electronic Design, Cambridge, UK), and recorded and stored on a personal computer using Signal 4.05b (Cambridge Electronic Design, Cambridge, UK).

At the start of each session, the motor hotspot (M1 hotspot) was identified as the coil position over the left M1 where a single magnetic stimulus elicited the largest MEP peak-to-peak amplitude in the relaxed right FDI. Once determined, M1 hotspot was marked on the subject’s scalp to ensure consistent coil positioning. Subsequently, the RMT was determined as the lowest intensity applied over M1 hotspot to evoke a response of more than 50 µV in the relaxed FDI in at least 5 of 10 trials [29]. TS intensity was adjusted as required to obtain MEP amplitudes of approximately 1 mV and used in all following TMS paradigms.

### TMS metrics for sensorimotor function

To assess the effect of ctDCS on inhibitory circuits in CD, the following TMS-based metrics were recorded before and after each ctDCS intervention: SICI, SAI, and CBI. Details are summarized in Table 1.

**Table 1:** Detailed TMS measurements

Paradigm	Conditioning stimulus (CS)	Test stimulus (TS)	Interstimulus interval (ISI)	EMG recordings	Trials
SICI	M1 80% RMT	M1 1mV MEP	2ms / 3ms	Right FDI	1 + 3 × 10
SAI	Right index finger 3x sensory threshold	M1 1mV MEP	25ms / 30ms / 40ms	Right FDI	1 + 4 × 10
CBI	Right cerebellar hemisphere 90% RMT	M1 1mV MEP	5ms / 6ms	Right FDI	1 + 3 × 10

*SICI* short-interval intracortical inhibition, *SAI* short-latency afferent inhibition, *CBI* cerebellar brain inhibition, *M1* primary motor cortex, *RMT* resting motor threshold, *MEP* motor evoked potential, *FDI* first dorsal interosseus muscle

1. Short-interval intracortical inhibition:

SICI was assessed using a paired-pulse TMS protocol in which two stimuli were delivered in rapid succession over the M1 hotspot using the same magnetic coil [30]. The CS was set at 80% of RMT [13] and preceded the TS by an ISI of 2 or 3 ms. For comparison, TS without CS were collected. Ten MEPs were recorded for each condition (2 ms, 3 ms, no CS) in randomized order.

2. Short-latency afferent inhibition:

SAI consisted of an electrical CS to the right index finger and a TS delivered by TMS to the left M1 [31]. The CS was delivered with ring electrodes strapped around the middle and distal phalanges of the right index finger, with the cathode placed proximal and the anode distal [32]. Stimulation was applied using a constant current stimulator (DS7A; Digitimer Ltd., Hertfordshire, UK). The stimulus had a duration of 0.1 ms and an intensity of three times of the patients individual sensory threshold. Stimuli were delivered at ISIs of 25, 30 or 40 ms. Ten responses were recorded for each condition (25 ms, 30 ms, 40 ms, no CS) in randomized order.

3. Cerebellar brain inhibition:

To assess CBI, a CS was applied over the right cerebellar hemisphere, followed by a cortical TS over M1 at ISIs of 5 or 6 ms. The intensity of the CS was set at 90% of RMT [12]. Three conditions were tested: TS alone and TS preceded by the CS at the two different ISIs. Each condition was repeated 10 times, presented in a randomized order.

### Cerebellar Transcranial Direct Current Stimulation (ctDCS)

CtDCS was delivered using a direct current stimulator (Eldith DC-Stimulator; NeuroConn GmbH, Ilmenau, Germany). For this purpose two 25 cm^2^ saline-soaked sponge electrodes connected to the stimulator were attached to the scalp as following: one electrode was placed 3 cm to the right of the inion, a second one on the right buccinator muscle [24]. A direct current of 2 mA was delivered for a duration of 20 min for anodal and cathodal stimulation, while in sham stimulation the direct current ceased after 30 s [33]. All three modes had a 8-second fade-in and fade-out to achieve optimal blinding between sessions [34].

### Data Analysis and Statistics

Recorded MEP amplitudes were exported as plain text. All MEP amplitude values were transformed to relative values with reference to the average amplitude of the TS in the same session and at the same time (pre / post ctDCS). The first trial of each series was discarded to avoid potential hyperexcitability related to the initial TMS impulse [35]. Trials showing pre-stimulus muscle activity exceeding 50 µV within 50 ms were excluded from the analysis [36]. Statistical analysis was performed using R software (version 4.4.2; R Foundation for Statistical Computing, Vienna, Austria). The R package ‘lmerTest’ was used for linear mixed-effects modeling [37] and ‘emmeans’ for post hoc contrasts and estimated marginal means [38].

RMT was analyzed to assess variation across sessions and potential impact of ctDCS interventions on motor cortical excitability using a linear mixed-effects model (LMM) with TIME (pre vs. post intervention) and SESSION (sham, anodal, cathodal ctDCS) as fixed effects and SUBJECT as a random effect.

For each TMS paradigm, the effect of the stimulation condition (CONDITION) was assessed using a LMM with MEP amplitude as the dependent variable, CONDITION as fixed effect, and SUBJECT as a random effect. Recordings prior to the ctDCS intervention from all three sessions were combined for this analysis to evaluate baseline effects. For SICI, CONDITION had three levels: TS alone, ISI 2 ms, and ISI 3 ms. For SAI, four CONDITION levels were used: TS alone, ISI 25 ms, ISI 30 ms, and ISI 40 ms. For CBI, CONDITION included three levels: TS alone, ISI 5 ms, and ISI 6 ms. In subsequent analyses, the CONDITION was only kept as a predictor if the results differed across levels; otherwise, the corresponding values were combined.

The effect of ctDCS was then modeled with MEP amplitude as the dependent variable, TIME, SESSION, and – if relevant – CONDITION as fixed effects, and SUBJECT as a random effect. TIME and SESSION were modeled with an interaction.

For the effect on motor symptom severity, TWSTRS was modeled with TIME and SESSION as fixed effects, an interaction between them, and SUBJECT as a random effect.

Predictors and 95% confidence intervals (95% CI) are reported. A p-value < 0.05 was considered statistically significant. Given the exploratory nature of the study, no correction for multiple comparisons was applied.

## Results

### Subjects

A total of 15 patients with isolated CD (13 females; median age 60 years [range 30–72]; median disease duration 6 years [range 1.5–25]) were included in this study. All patients were right-handed according to the Edinburgh Handedness Inventory and completed the three experimental sessions without any adverse effects during or after stimulation. Demographic and clinical characteristics of the patients are shown in Table 2.

**Table 2:** Demographic and clinical characteristics of the subjects

Patient No.	Age (y)	Sex	Disease duration (y)	TWSTRS	Clinical characteristics
1	66	f	7	17	Torticollis to the left, laterocollis to the left, anterocollis, sagittal shift to the front
2	69	m	7	16	Torticollis to the right, retrocollis, sagittal shift to the front
3	39	f	6	3	Retrocollis
4	66	f	6	22	Torticollis to the right, shoulder elevation right side
5	46	f	6	5	Laterocollis to the right, retrocollis
6	63	f	5	19	Torticollis to the left, retrocollis, sagittal shift to the front
7	72	f	8	12	Torticollis to the right, shoulder elevation right side
8	57	f	6	4	Torticollis to the right
9	48	f	1,5	5	Torticollis to the left, retrocollis, sagittal shift to the front
10	44	f	15	6	Torticollis to the right, retrocollis
11	30	m	15	5	Laterocollis to the left, anterocollis
12	69	f	3	24	Laterocollis to the left, shoulder elevation left side, retrocollis
13	60	f	4	20	Torticollis to the left, laterocollis to the right
14	50	f	8	17	Torticollis to the left, laterocollis to the right, retrocollis
15	71	f	15	6	Laterocaput to the left

*TWSTRS* motor subscore of the Toronto Western Spasmodic Torticollis Rating Scale (prior to the first experimental session), *m* male, *f* female

### RMT

RMT estimates across SESSIONs and TIME ranged between 43.8% and 45.7% (Table 3). SESSION (*p* = 0.548) and TIME (*p* = 0.974) had no significant effects on RMT.

**Table 3:** RMT across intervention modes

Parameters	Sham ctDCS		Anodal ctDCS		Cathodal ctDCS	
	Estimate	95% CI	Estimate	95% CI	Estimate	95% CI
RMT pre (%)	43.8	40.2–47.4	45.7	42.0–49.3	44.1	40.5–47.8
RMT post (%)	44.5	40.9–48.2	44.7	40.8–48.1	44.7	41.0–48.3
ΔRMT (post – pre)	0.7	-1.1–2.5	-1.2	-3.0–0.6	0.5	-1.3–2.3

Average values are represented by model estimates; error ranges indicate 95% confidence intervals (95% CI). *RMT* resting motor threshold, *ΔRMT* change in RMT from pre- to post-intervention, *ctDCS* cerebellar transcranial direct current stimulation

### SICI

**Fig. 2:**
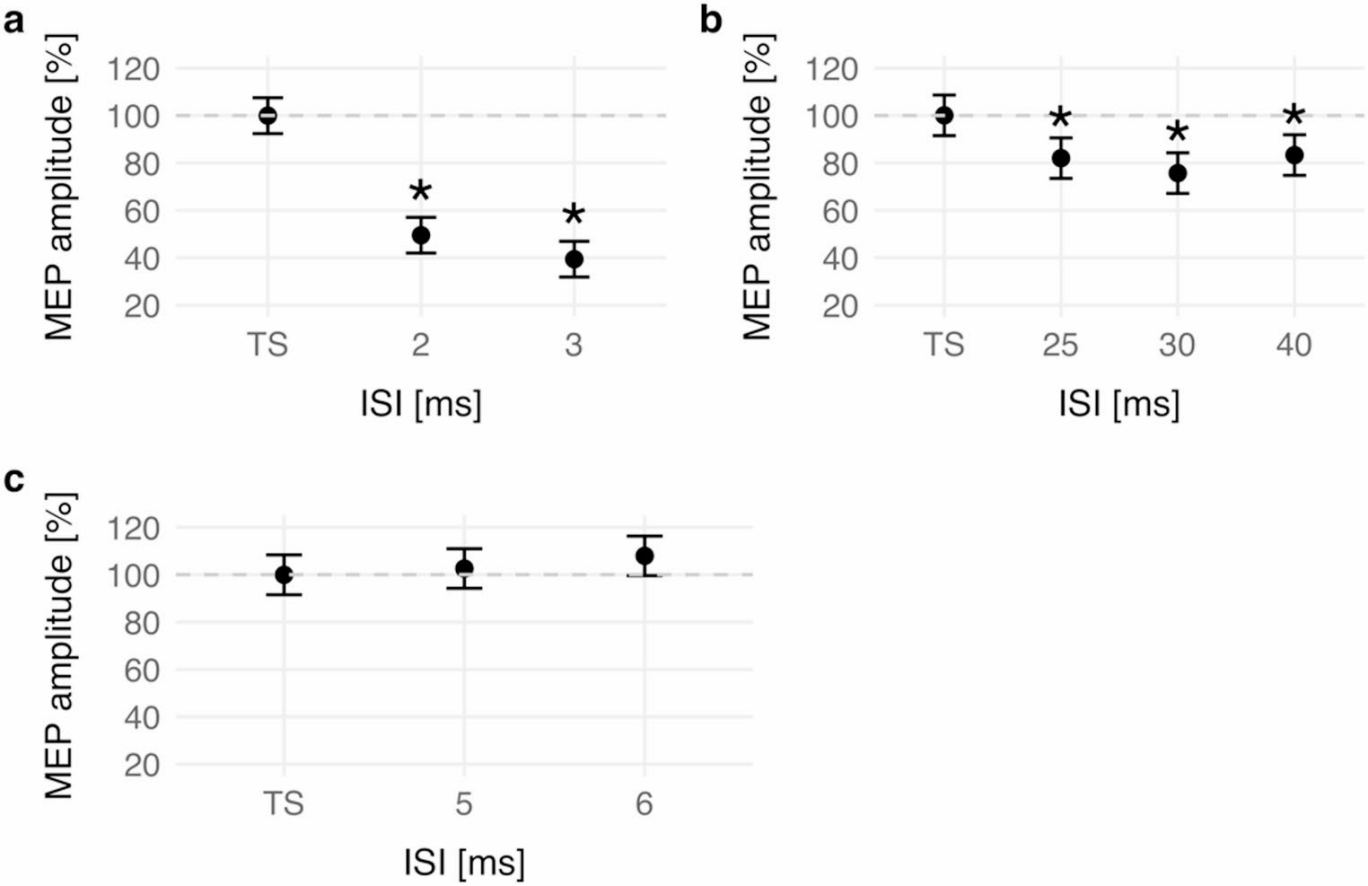
Baseline analysis of TMS paradigms. MEP amplitudes for different CONDITIONs are illustrated as relative amplitudes in comparison to the TS. Error bars represent the 95% CI; asterisks indicate a significant reduction of MEP amplitudes in comparison to TS alone. (**a**) In the SICI paradigm, both 2 ms and 3 ms ISIs lead to a significant suppression of MEP amplitudes. (**b**) In SAI, MEP amplitudes were significantly suppressed at ISIs 25 ms, 30 ms and 40 ms. (**c**) In CBI, baseline analysis revealed no significant effect of CONDITION. *MEP* motor evoked potential, *ISI* interstimulus interval, *TS* test stimulus

In the pre-interventional baseline series, CS reduced MEP amplitudes significantly (*p* < 0.001). Post hoc comparisons demonstrated that SICI with an ISI of 2 ms reduced MEP amplitudes to 49.5% [41.6%-57.4%] (*p* < 0.001), and an ISI of 3 ms reduced MEP amplitudes to 39.4% [31.4%-47.3%] (*p* < 0.001) compared to TS alone. MEP suppression was significantly stronger at an ISI of 3 ms than at 2 ms (*p* = 0.008) (Fig. 2a).

**Fig. 3:**
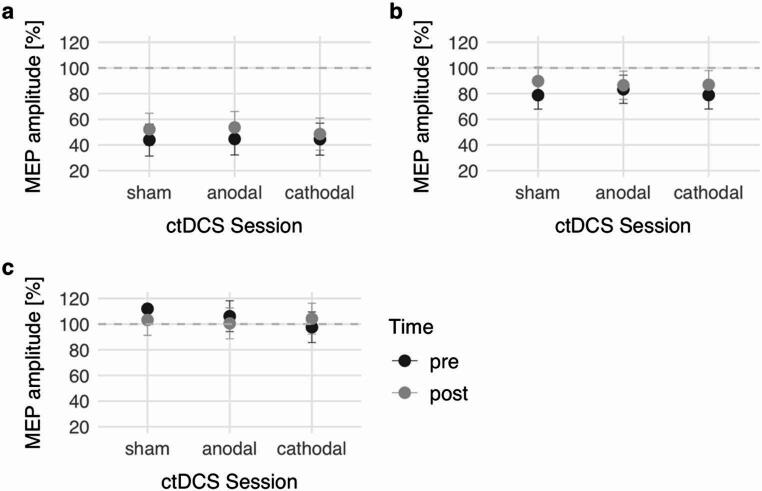
Effect of ctDCS interventions on TMS paradigms. MEP amplitudes are illustrated as relative amplitudes in comparison with the TS alone. Error bars represent the 95% CI. (**a**) SICI paradigm, (**b**) SAI paradigm, (**c**) CBI paradigm. Different intervention modes (sham, anodal and cathodal ctDCS) did not significantly modulate MEP amplitudes in any of the TMS paradigms. *MEP* motor evoked potential, *ctDCS* cerebellar transcranial direct current stimulation

Comparing the ctDCS sessions, there was a significant main effect of TIME (*p* = 0.002) and CONDITION (*p* < 0.001), but no main effect of SESSION (*p* = 0.647) and no TIME x SESSION interaction (*p* = 0.634) (Fig. 3a).

### SAI

In baseline analysis, there was a significant main effect of CONDITION, indicating that SAI led to a significant reduction of MEP amplitudes compared to TS alone (*p* < 0.001). Post hoc testing revealed MEP amplitudes were suppressed to 82.0% [73.3%-90.7%] (*p* = 0.002), 75.7% [66.9%-84.4%] (*p* < 0.001), and 83.3% [74.5%-92.1%] (*p* = 0.005) at the ISIs of 25 ms, 30 ms, and 40 ms, respectively. There were no differences in MEP inhibition between the different ISIs (Fig. 2b).

Further intervention-related analysis demonstrated a significant main effect of TIME (*p* = 0.016), but no main effect of SESSION (*p* = 0.857). Again, there was no TIME x SESSION interaction (*p* = 0.570) (Fig. 3b).

### CBI

For CBI, baseline analysis revealed no significant effect of CONDITION (*p* = 0.281), reflecting comparable MEP amplitudes at ISIs of 5 ms, 6 ms, and TS alone (Fig. 2c).

Intervention-related analysis showed no main effects of TIME (*p* = 0.522) or SESSION (*p* = 0.370) and no TIME x SESSION interaction (*p* = 0.242) (Fig. 3c).

### Clinical Symptom Severity

**Fig. 4:**
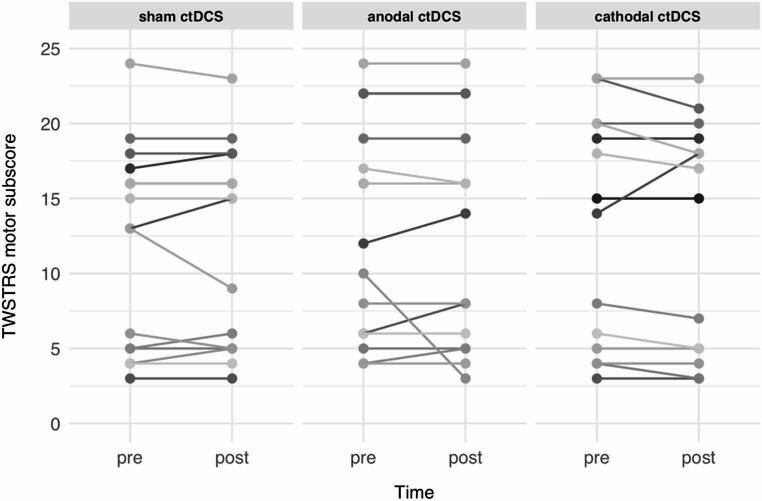
Individual TWSTRS motor subscore of all patients before (pre) and after (post) each of the three sessions. Every set of lines in a particular shade of grey refers to a single patient. The figure illustrates that no significant change of motor symptom severity occurred within or across sessions. *TWSTRS* Toronto Western Spasmodic Torticollis Rating Scale, *ctDCS* cerebellar transcranial direct current stimulation

Averaged across all sessions and all patients, TWSTRS motor subscore at baseline (prior to the first experimental session) was 12.3 [range 3–24] before and 12.1 [range 3–24] after intervention (Fig. 4). LMM analysis for TWSTRS revealed no main effect of TIME (*p* = 0.646) or SESSION (*p* = 0.253), and no TIME x SESSION interaction (*p* = 0.977).

## Discussion

In the present study, we did not detect an influence of anodal or cathodal ctDCS on the investigated inhibitory circuits of the sensorimotor network or on clinical symptom severity. Based on these results, the neurophysiological mechanisms of a single session ctDCS on motor network excitability and connectivity in isolated CD remain unclear.

### Baseline Analysis of Inhibitory Circuits

SICI, reflecting GABA_A_-mediated intracortical inhibitory mechanisms [39], was present in our patient cohort at both ISIs investigated, suggesting preserved activity of inhibitory interneurons within M1. This finding is consistent with the fact that most previous studies report intact SICI in patients with CD [11, 13], while only few studies show abnormal SICI in CD patients [12].

SAI, a marker of sensorimotor integration involving cholinergic and GABA_A_-mediated cortical networks [40], also showed preserved inhibition in our cohort at baseline across all ISIs. Similar to SICI, previous reports on sensorimotor integration in CD are conflicting. While some studies have reported normal SAI in CD [41, 42] consistent with our results, others indicated an impairment [40, 43].

The variability and sometimes conflicting findings regarding both SICI and SAI may be explained by small sample sizes, heterogeneous patient groups, and methodological variability, such as varying ISIs and different intensities of TS and CS, which complicates direct comparisons between study populations.

Regarding CBI, cerebellar conditioning in our cohort had no inhibitory effect on MEP amplitudes at either 5 or 6 ms ISI, supporting that CTC connectivity in CD is impaired. This is in line with previous studies reporting reduced or absent CBI in CD patients [12, 13]. Nevertheless, contrasting findings have also been reported, as some studies demonstrated intact CBI in patients with CD [11, 44]. These discrepancies in the literature may be explained by variability in patient characteristics, particularly symptom severity. Sondergaard et al. demonstrated that CBI variability is high, even among healthy individuals, and that reduced CBI correlates with motor symptom severity in CD, suggesting that studies including patients with different severity levels can lead to inconsistent results [44]. In addition, differences in coil type and placement may also contribute to heterogeneity across studies. Some studies using double-cone coils reported a more effective activation of cerebellar circuits compared to figure-of-eight coils, suggesting that coil geometry and stimulation depth are relevant factors for eliciting CBI [45, 46]. Nevertheless, figure-of-eight coils have also been applied in studies investigating CTC interactions, including CBI investigations in CD. Notably, Brighina et al. [13] and Porcacchia et al. [12] both reported significant alterations in CBI in CD patients using this coil type. Additional studies have further demonstrated that figure-of-eight coils can reliably modulate cerebellar excitability [11]. In addition, the use of double-cone coils may be associated with increased discomfort, potentially affecting the tolerability of the stimulation and the data quality [47, 48]. Overall, the optimal coil type for cerebellar stimulation remains a matter of ongoing debate and should be considered when comparing findings across studies.

### Effects of ctDCS Interventions

When analyzing the effects of anodal and cathodal ctDCS on different neurophysiological parameters of the sensorimotor network, we observed no significant modulation of the inhibitory circuits of SICI, SAI, or CBI compared to sham stimulation. Of note, there was an effect of TIME on MEP amplitudes in the SICI and SAI recordings, which points to changes of MEP amplitudes within sessions. These findings most likely reflect temporal variability in sensorimotor excitability, as long-lasting TMS experiments are known to be sensitive to several confounding factors including the patients attention, circadian effects, and repeated testing over time [28, 49, 50]. Given that our experimental sessions lasted approximately 2–3 h, such time-dependent effects are plausible. Importantly, the absence of a significant TIME x SESSION interaction indicates that these intra-session differences were not specific to the neuromodulatory effect of ctDCS.

The absence of RMT modulation in our study cohort aligns with previous studies showing no measurable influence of ctDCS on corticospinal or motor cortex excitability in either healthy participants or patient populations, with effects appearing to be specific to cerebello-cortical connections [23, 24]. Similarly, SICI remained unchanged following any of the ctDCS interventions. This finding is in line with previous studies in both healthy participants [24] and CD patients [11, 12], which consistently showed no modulation of intracortical inhibition using either ctDCS or cerebellar continuous theta burst stimulation (cTBS), indicating that modulation of cerebellar excitability has no direct influence on local inhibitory circuits within M1.

Another important finding is that ctDCS did not modulate SAI in our patients. This is notable given previous studies showing that cerebellar NIBS can alter sensorimotor plasticity investigated by PAS [5, 51, 52]. Doeltgen et al. proposed that the cerebellum modulates PAS but not SAI because PAS primarily depends on NMDA-receptor-mediated plasticity, whereas SAI is mediated by cholinergic and GABA_A_-ergic circuits, which may be less sensitive to cerebellar modulation [53]. Our findings support the notion that SAI and PAS engage distinct afferent networks with different susceptibilities to cerebellar NIBS.

Likewise, CBI was not modulated by ctDCS in our patients. As ctDCS has been shown to influence CBI in healthy individuals [24], our findings suggest that the CTC pathway in CD may be less responsive to neuromodulation. This is consistent with the proposed impairment of cerebellar-M1 connectivity in CD. In line with this hypothesis, Porcacchia et al. showed that cerebellar cTBS modulated CBI in healthy individuals, but had no effect in CD patients [12]. Notably, there is conflicting evidence from a small study that applied bilateral cerebellar cTBS in CD patients repeatedly over two weeks and reported a modulation of CBI. However, in the dissenting study, the observed reduction in CBI occurred at an ISI of 10 ms rather than at the frequently assessed interval of 5–7 ms associated with cerebellar inhibition, indicating that different mechanisms may have contributed to this effect [11].

Consistent with the absence of neurophysiological changes, we observed no improvement in clinical symptom severity following a single session of anodal or cathodal ctDCS, as assessed by the TWSTRS motor subscore. This finding aligns with the majority of previous studies that failed to demonstrate clinical benefits from a single session of cerebellar stimulation in dystonia patients [5, 54]. The most notable clinical improvement in CD was reported by Koch et al., who observed an improvement of approximately 15% in TWSTRS after a two-week stimulation protocol using bilateral cerebellar cTBS [11]. Although improvements following ctDCS have also been reported in FHD, for example in terms of handwriting kinematics [55], these findings are based on a small sample size (*n* = 8) and task-specific outcome measures. Moreover, as they were obtained in FHD patients, they may not be generalizable to CD and other dystonia subtypes, as the current literature does not allow for definitive conclusions regarding the extent to which CD and FHD share identical or distinct pathophysiological mechanisms. Both CD and FHD are considered network disorders involving distributed motor and sensorimotor circuits, sharing key pathophysiological features including impaired inhibitory control and abnormal sensorimotor integration [6, 7, 56]. However, as some variability in neurophysiological findings across studies has been reported, this may reflect heterogeneity across dystonia phenotypes [57]. Recent studies have therefore emphasized that focal dystonias may potentially differ in the relative contribution or organization of network abnormalities [58–60]. Based on this consideration, it may be plausible that responsiveness to cerebellar neuromodulation differs between dystonia subtypes.

Overall, current studies provide no support for clinically relevant benefits of a single session of ctDCS in CD. Future work should therefore investigate alternative stimulation protocols, such as repetitive stimulation sessions, to induce cumulative and longer-lasting effects through modulation of cortical plasticity [61], since similar approaches have induced promising effects in other neurological disorders [62, 63]. Bifocal stimulation protocols also need to be considered, e.g. bilateral ctDCS or tDCS targeting both the cerebellum and M1, as these have already been applied successfully in healthy participants and in Parkinson`s disease patients [64, 65].

### Limitations

Several methodological details need to be considered in this study. The inclusion of a healthy control group may have improved the interpretation of baseline findings in terms of SICI, SAI, and CBI in our CD cohort. Furthermore, the heterogeneity of the patient cohort in terms of age, disease duration, and clinical symptom severity may have reduced the ability to detect subtle neurophysiological or clinical changes. In addition, the study may have been underpowered to identify small-to-moderate effects, although small sample size is a common limitation in studies on CD. Also, tDCS protocols targeting the motor cortex have demonstrated high interindividual variability in neurophysiological responses [15], which may also apply to cerebellar stimulation and contribute to variable outcomes. Additionally, the complex structure and architecture of the cerebellum pose a fundamental challenge for ctDCS, leading to the fact that the precise mechanism of ctDCS at the cellular level remains incompletely understood [66].

## Conclusion

In conclusion, our study indicates that a single session of ctDCS has no potential to influence altered network connectivity and clinical symptoms in isolated CD patients. Our results highlight the need for future research to focus on repetitive ctDCS interventions or multifocal stimulation protocols, which may be required to induce meaningful effects in sensorimotor network excitability and clinical symptom severity.

## Data Availability

Data available on reasonable request from the corresponding author.
